# Palliative care for patients with substance use disorder and multiple problems: a qualitative study on experiences of healthcare professionals, volunteers and experts-by-experience

**DOI:** 10.1186/s12904-019-0502-x

**Published:** 2020-01-14

**Authors:** Anne Ebenau, Boukje Dijkstra, Chantal ter Huurne, Jeroen Hasselaar, Kris Vissers, Marieke Groot

**Affiliations:** 1 Department of Anesthesiology, Pain and Palliative Care, Radboudumc Expertise centre for Pain and Palliative Medicine, Internal Post 549, Radboud University Medical Centre (Radboudumc), P.O. Box 9101, 6500 HB Nijmegen, The Netherlands; 2 Nijmegen Institute for Scientist-Practitioners in Addiction (NISPA), Postbus 9104, 6500 HE Nijmegen, The Netherlands; 3 Salvation Army, Central Netherlands, Zandvoortweg 211, 3741 BE Baarn, The Netherlands; 4 Tactus Addiction Care, Lokatie Ripperdastraat, Ripperdastraat 8, 7511 JR Enschede, The Netherlands

**Keywords:** Qualitative study, Palliative care, End-of-life, Terminal care, Substance use disorder, Addiction, Healthcare professionals

## Abstract

**Background:**

There is little information about how healthcare professionals feel about providing palliative care for patients with a substance use disorder (SUD). Therefore, this study aims to explore: 1) the problems and needs experienced by healthcare professionals, volunteers and experts-by-experience (HCP/VE) during their work with patients with SUD in a palliative care trajectory and; 2) to make suggestions for improvements using the quality of care model by Donabedian (Structure, Process, Outcome).

**Methods:**

A qualitative study was conducted, consisting of six focus group interviews which consisted of HCP/VE working with patients with SUD in a palliative care phase. At the end of the focus group interviews, participants structured and summarized their experiences within a Strengths, Weaknesses, Opportunities and Threats (SWOT) framework. Interview transcripts (other than the SWOT) were analysed by the researchers following procedures from the Grounded Theory Approach (‘Grounded Theory Lite’). SWOT-findings were not subjected to in-depth analysis.

**Results:**

HCP/VE stated that within the Structure of care, care networks are fragmented and HCP/VE often lack knowledge about patients’ multiplicity of problems and the time to unravel these. Communication with this patient group appears limited. The actual care-giving Process requires HCP/VE a lot of creativity and time spent seeking for cooperation with other caregivers and appropriate care settings. The latter is often hindered by stigma. Since no formalized knowledge is available, care-delivery is often exclusively experience-based. Pain-medication is often ineffective due to active substance use. Finally, several Outcomes were brought forward: Firstly, a palliative care phase is often identified only at a late stage. Secondly, education and a (mobile) team of expertise are desired. Thirdly, care for the caregivers themselves is often de-prioritized.

**Conclusions:**

Better integration and collaboration between the different professionals with extensive experience in addiction, palliative and general curative care is imperative to assure good palliative care for patients with SUD. Currently, the resources for this care appear to be insufficient. Development of an educational program and social mapping may be the first steps in improving palliative care for patients with severe SUD.

## Background

People with a substance use disorder (SUD) are likely to develop more chronic and life-threatening conditions and die earlier than the general population [1–7]. Although most people with SUD recover, others may suffer from relapse or have lifelong addiction problems [8, 9]. With regard to this last group and in view of their ever-decreasing health and wellbeing, they may benefit from palliative care (PC). However, it is surprising that both evidence and expertise on PC for patients with SUD is virtually non-existent.

Studies show that the little attention that there is for PC for patients with SUD, mainly focuses on ‘prescription drug abuse’, dependence-producing medicines (mainly opioids), pain management, and alcohol abuse within PC phases [10–14]. Furthermore, reports and studies about patients with pre-existing SUD in a PC phase often lack attention to the psychological, social and spiritual domains and are short on scientific empirical rigor. They are based on one case, are non-replicable or reflect only authors’ opinions [13, 14]. Such literature demonstrates that factors, such as, non-compliance to treatment, symptom expression and representation, non-disclosure of substance use, and stigma jeopardize good PC for patients with SUD. There is especially a need for more knowledge, financial resources and non-judgmental attitudes of healthcare professionals, and better assessment of substance use and symptoms. Pain is often under treated and medical treatment remains insufficient [15–24].

Patients with SUD often have multiple problems, e.g. psychiatric co-morbidity, homelessness and intellectual disability [25–28]. Fortunately, the body of literature on patient groups with these problems is increasing, however, shows similar problems in the provision of PC. In addition, such care suffers from ethical issues surrounding decision-making and lack of resources, care-coordination and appropriate care-settings. Hence, creativity, consultation and education are needed [29–33]. These issues might account for PC for patients with SUD as well.

The lack of sound research and literature about palliative care for patients with SUD and multiple problems (SUD+), is a major shortcoming. Therefore, we performed an explorative, qualitative, study aiming to investigate the problems, needs and possible improvements suggested or experienced by healthcare professionals, volunteers and experts-by-experience (patients with lived experience of SUD, however, in recovery) regarding PC for patients with SUD+.

## Methods

### Study design

This qualitative study includes six, single focus group interviews with Dutch healthcare professionals, volunteers and experts-by-experience (HCP/VE) about PC for patients with SUD+. At the end of the interviews, participants structured and summarized their experiences in a Strengths, Weaknesses, Opportunities and Threats (SWOT) framework [34]. Interview transcripts (except the SWOT) were analyzed using elements of the Grounded Theory Approach (Grounded Theory ‘lite’) [35, 36]. Data collection lasted from September till October 2017. The COREQ (COnsolidated criteria for REporting Qualitative research) checklist was used to guide this publication [37]. An extensive description of the methodology and design of this study has been published previously [13].

### Participant recruitment

We used purposive sampling [38, 39] to ascertain a diversity of HCP/VE from addiction, palliative and general curative healthcare (neither specialized in palliative nor addiction care). Table 1 provides information about the organization of different types of care in the Netherlands [40, 41]. Such purposive sampling, furthermore, enabled inclusion of participants from different regions of the Netherlands (south, middle and western parts), which was needed to grasp a potential variety in the organization of PC for patients with SUD+ within the country. Different recruiting strategies were described in the protocol article of this study [13]. Refusal of participation was not counted.

**Table 1:** Organization of addiction and palliative care and the Salvation Army (the Netherlands)

Care	Organization
Addiction care	The majority of regular substance use treatment centers provides the entire range of addiction treatment, from prevention to maintenance treatment, from outpatient (80%) to clinical admissions. On average, each institution employs around 1000 people and treats around 9000 patients per year. Some of these institutions are integrated into mental health care. Most institutions (*n* = 12) are united in a network with two research institutes, a network of client representation boards, and the sector organization of mental health (The Dutch Addiction Association). Treatment methods include motivational interviewing techniques, cognitive behavioral therapy, community reinforcement approach, Minnesota Model, medication, and e-health. Care focuses on psychosocial, physical and medical level. Co-morbid disorders are treated as much as possible at the same time. For specialist care, other than addiction, patients are referred.
Palliative care	One of the main PC principles in the Netherlands is that nearly all professional HCPs must be able to give basic PC. It is part of regular, generalist care. In complex situations, due to the amount, variety and interaction of problems and/or due to lack of knowledge and experience, a broad expert network of professionals is available. Often they work together in Palliative Care Consultation Teams (PCC teams). These teams mostly don’t take over care, but stay in an advisory role towards the principle care providers. In case expert palliative care is needed constantly, PC units in hospitals, hospices and nearly-at-home-houses are available. Terminal inpatient care is also possible there. Next to experienced and expert HCP, well-trained volunteers are invaluable in many care settings. They support patients and their informal caregivers to give room to relieve in the last phase of life.
Salvation Army	More than hundred years ago, the Salvation Army started her activities in the Netherlands. Nowadays, this organization offers social care, elderly- and healthcare, mental healthcare, child welfare, addiction care, prevention, social reintegration and rehabilitation work. Also, Salvation Army aims to be actively present on a local level, e.g. by offering neighborhood activities or church services. In 2017, 108.275 people got in touch with Salvation Army’s activities. The same year, almost two million nights in shelters or other accommodations were arranged, of which 80% was covered by homeless people. Over six million meals were served within community centers and shelters or via ‘soup busses’ for homeless people and temporary accommodation for refugees. 13.000 volunteers and around 6500 employees work for the Salvation Army. Inspired by the spirit of God, they aim to be of service. In their vision, every human matters and deserves to be there.
Volunteers	Volunteers are active in both palliative and addiction care and in the Salvation Army. In 2017, most were aged between 35 and 45 and, on average, volunteered 4,5 h a week. The same year, almost half of the Dutch population volunteered once a year.

Inclusion criteria for participating HCP/VE were: 1) being 18 years or older; 2) speaking Dutch well-enough and; 3) having experience with at least one patient with SUD+ in a palliative care phase. The third criterion was set low, because the patient group seems to be rather small, although official estimates are unknown [42]. Also, the researchers believed that caring for these patients makes a great impact and as a consequence, HCP/VE are well-aware of their experiences and well-capable in recapitulating these. We focused on PC for patients with severe SUD (six or more symptoms, on DSM-V criteria [43]) and multiple problems, suffering from a somatic, irreversible life-threatening disease/co-morbidity or from severe physical deterioration as a result of ongoing, chronic SUD without the prospect of cure [13].

### Data collection

Focus group interviews were chosen as the method of data collection, because it is time-efficient and suitable for collecting a great amount of and variety in experiences from the participants. We aimed to create rich inter- and multidisciplinary interaction and discussions [44]. The semi-structured interview guide was based on the literature and the experience of the project group. It was developed and peer-reviewed together with experts-by-experience (a translated version of the interview guide can be found in Additional file 1). This guide allowed the group-facilitators to explore, prompt and explicate HCP/VE opinions and actual care-experiences [38, 44]. The guide addressed the following main topics: 1) Physical, social, psychological, existential aspects of care; 2) Organization of PC; 3) Communication with patients; 4) Care for proxies and; 5) HCP/VE knowledge and feelings of competence in caring for this patient group.

Before a focus group interview started, HCP/VE were asked for their profession, work setting, years of work experience, self-reported competence in SUD and PC, and mean number of patients with SUD+ with PC needs they cared for. Each focus group interview ended with a practical SWOT-session: HCP/VE were asked to write notes, brief and to the point, concerning Strengths, Weaknesses, Opportunities and Threats on the main topics [34].

Each focus group interview lasted approximately 2 h and was led by group-facilitators who are experienced qualitative researchers (MG, AE, JH and YE (see Acknowledgements)). During the focus group interviews, the head researcher (AE) made field notes.

### Data analysis

The head researcher (AE) listened to all of the focus group interviews to familiarize herself with the data. We did not aim at theory development, however, used several procedures of the Grounded Theory Approach for the analysis of the interview transcripts (except for the SWOT); i.e. Grounded Theory ‘lite’. This inductive or data-driven method was chosen because of its explicitly open and detailed character [35, 36]. First, in-vivo codes, relevant to the research question, were connected to words or text segments of the first focus group interview. Over**-**interpretation was prevented by staying close to the original data. Inter-coder reliability between the two researchers (AE and MG) was high on the first focus group interview and therefore, open coding on the second focus group interview was done by AE alone. Next, AE clustered these open codes into exclusive (meaning: internal homogeneity and external heterogeneity) sub-codes, main-codes and themes, which were connected (axial coding) and brought these together in a descriptive codebook, serving as a coding strategy for the remaining focus group interviews (selective coding). The codebook was refined in the process of collecting and analyzing data (using the constant comparative method) [35, 36, 45] and extensively discussed by AE and MG. After coding four focus group interviews with the codebook, no new codes emerged and data saturation was reached [46]. The analysis of the remaining two focus group interviews helped gaining deeper understanding of the ‘essence’ of the themes and codes which - in the final step – were all incorporated into and thus covered within the quality of care model by Donabedian (Structure, Process, Outcome) [47, 48]. We added the heading ‘Patient characteristics’. Analysis was supported by ATLAS.ti 8.0.34 research-software. A member check was not performed due to time-restraints.

Set apart from this analysis, were the data, i.e. notes collected from the SWOT-session. These notes were structured by AE, however, not subjected to an in-depth analysis. Both in the focus group interviews as well as in this article, the SWOT-framework is used to summarize findings and provide true insight into clinical practice. Eventually, the SWOT-framework as presented in this paper (Fig. 2) contains both in-depth findings from the focus group interviews and the content of the SWOT-sessions at the end of these interviews.

## Results

### Participant characteristics

In total, 48 healthcare professionals and four volunteers (including one expert-by-experience) were included, which made up for a multidisciplinary composition (Table 2). The majority of HCP/VE were women. The mean age was about fifty years old. On average, the participants had cared for or supported eight patients with SUD+ in a PC phase during the last 3 years (SD = 8.2, median = 5, range 1 to 40; two participants were removed from this calculation as they stated to have supported three hundred patients). Furthermore, most HCP/VE worked at ‘in-patient’ settings and one-third worked for the Salvation Army. Self-reported competency on PC and addiction differed between HCP/VE from different settings.

**Table 2:** Characteristics of HCP/VE

Participants				52
Mean age in years (SD)				49.7 (12.8)
Gender				
Male				14 (27%)
Female				38 (73%)
Occupation				
Nurse (practical nurse, general nurse, nurse practitioner)^a^				23 (44%)
Social worker / personal carer (not medically trained)				6 (12%)
General Practitioner				4 (8%)
Addiction physician				4 (8%)
Volunteer				4 (8%)
Pain specialist (anesthesiologist) consultant in palliative care				2 (4%)
Psychologist				2 (4%)
Other, e.g. spiritual worker, care-coordinator, psychiatrist				7 (13%)
Setting				
Addiction or psychiatry				21 (40%)
Palliative or terminal care				16 (30%)
Both				12 (23%)
General or other care				3 (6%)
Experience in current profession in years (mean)				11.4
Mean number of patients with SUD in palliative care phase cared for / supported in past 3 years				
1–10				39 (75%)
11–20				8 (15%)
21–30				2 (4%)
31–40				1 (2%)
300				2 (4%)
Self-reported competency in addiction per setting^b^				
	Addiction or psychiatry	Palliative or terminal care	Both	General or other care
Mean (SD)	7.5 (0.9)	5.9 (1.6)	7.3 (1.2)	5.8 (0.3)
Self-reported competency in palliative care per setting^c^				
	Addiction or psychiatry	Palliative or terminal care	Both	General or other care
Mean (SD)	6.2 (1.8)	7.8 (1.0)	7.3 (1.4)	6.5 (2.3)

^a^Respective education levels of these participating nurses are: secondary vocational education; university of applied science; university; ^b^Theoretical range: 1–10; ^c^Idem

### Qualitative findings

**Fig. 1:**
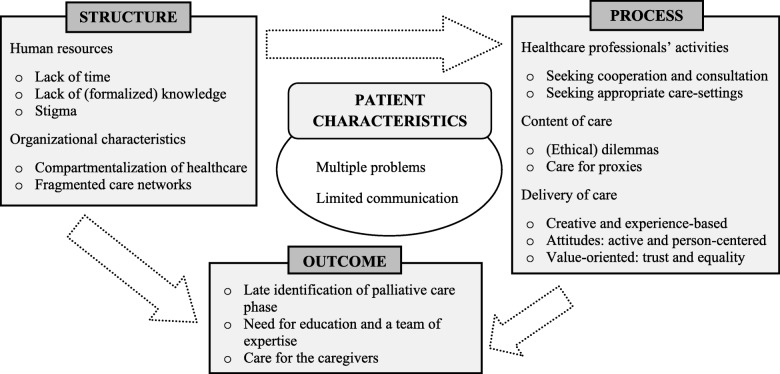
Quality of care model: Palliative care for patients with substance use disorder and multiple problems

Figure 1 shows four components of PC for patients with SUD+. The central circle is an addition to the Donabedian model and shows Patient characteristics, which contribute to the complexity of caring for this patient group. Second, under Structure we find the more factual circumstances of the settings in which care is provided. Third, under Process we find key terms for daily practice: the care actually received by patients and offered by HCP/VE. Fourth, the effects of Structure and Process are listed under Outcome.

### Patient characteristics

#### Multiple problems

Compared to more ‘regular’ or ‘general’ patients, HCP/VE agreed that patients with SUD+ often have more, as well as more complex problems. Unfinished business, loneliness and shame are more often at play. HCP/VE refer to these as “raw” problems. Psychiatric co-morbidities are also mentioned. Physically, patients differ with regard to a higher frequency of particular diseases (e.g. COPD and cancer of the throat) and symptoms, craving for substances and early death. Assessment of physical, psychosocial and existential needs is hindered by a cumbersome communication between patients and HCP/VE.

*They know how to make a good impression, while actually, there are so many problems on all dimensions. There are people of whom you think ‘he will last a while’ and then, they suddenly die. It is also the complexity of problems.*

- Female, group III (central part of the Netherlands)

#### Limited communication

On the one hand, HCP/VE stated that patients keep quiet about or underreport substance use. In such cases, HCP/VE fail to understand certain phenomena, e.g. why pain medication is ineffective. All the more this is so when patients are intoxicated: patients then, could experience (and express) their symptoms or body contact differently or not at all. On the other hand, HCP/VE mentioned that conversations are dominated by patients asking for substances (drugs, alcohol or medicines) or manipulative behavior.

*… there will always be addiction behavior. And you have to take that into account when people ask for more morphine.*

- Male, group II (central part of the Netherlands)

Sometimes, anger, distrust or psychiatric co-morbidity play a role in how interaction takes place.

*You did everything on your own and now, suddenly, everybody is there to help. On the streets, however, you can’t trust everybody who may be there to help you.*

- Female, group II (central part of the Netherlands)

Patients can be hesitant and elusive in talking about death and dying as they might be suffering deeply. Some patients live in ‘the here and now’ which complicates conversations about the future.

*Partly, it is self-protection as well [..] they are deeply disappointed in their social environment [..]. They built a shell around themselves and are like ‘no!’.*

- Female, group V (southern part of the Netherlands)

Open and in-depth communication and assessment of problems is even more hindered by the fact that some patients are intellectually disabled or cognitively damaged and have difficulty expressing themselves. Also, HCP/VE mentioned unclear medical histories, lack of contact persons and far-progressed symptoms as a result of patients’ care avoidance.

**Female:** *They are the care avoiders, right?* **Female:** *Exactly. First, it all comes crashing down. And then, thinking back, you can find out what went wrong.*

- Group I (southern part of the Netherlands)

### Structure

#### Human resources

HCP/VE stated to be frustrated about the context in which PC for patients with SUD+ is provided and suggested that governments, managers and healthcare insurances should be motivated to further develop integrated PC for this patient-group. Shortage of time was perceived as distressing, because it prevented building trust and the unraveling of patients’ multiple problems. Volunteers and chaplains, however, stated to have more time for the psychological and existential dimensions.

*Without trust, you can’t treat a patient like this. It is the first thing. […] it takes time.*

- Male, group IV (southern part of the Netherlands)

Furthermore, it was pointed out that psychiatry is the primary focus of addiction care, and that there is less knowledge on and attention for the physical dimension and PC. HCP/VE from this field often associate PC with terminal care. In return, PC professionals master the somatic dimension and are looking from a multidimensional perspective, but know little of addiction care. Exchange of knowledge is desired to remedy the fragmentation. Only few healthcare professionals master both specializations. Existent protocols and (dosing) guidelines often do not meet the PC needs of patients with SUD+.

Also, HCP/VE stated that, in contrast to their own dedication to this patient group, other people are prejudiced, non-understanding and moralizing. As a consequence, patients feel unwelcome in many places. Stereotypes lie at the base of policies and attitudes, which hinders the provision of person-centered and empathic care.

*In society there’s this stigma: ‘it is your own fault, why should I spend my time on you?’. [...] They don’t look at what life somebody had and how he ended up like this.*

- Female, group IV (southern part of the Netherlands)

#### Organizational characteristics

HCP/VE explained that when this patient group receives inpatient care from one institution, other institutions and their expertise often are excluded, because insurance and reimbursements are tied to one institution. Such compartmentalization could result in healthcare professionals, who became involved because of their expertise, were working in their private time. With regard to the multiplicity of problems experienced by the patients, however, cooperation is necessary.

The organizational structure of the care itself appears fragmented. HCP/VE in addiction care and those in PC do not know each other or do not know how and when to contact each other. HCP/VE reported that external expertise is not used as it should be. Especially the input of the addiction specialist is under-used. Division of responsibilities is unclear too. Social mapping and integration within existing networks could clarify the organization of PC for patients with SUD+.

### Process

#### Activities of HCP/VE

HCP/VE agreed upon the importance of and willingness to improve cooperation. Currently, contact between addiction, palliative and general care is neither proactive nor structural. Good cooperation and consultation should be multidimensional, timely and ongoing. It needs motivated people and efficient information transfer between institutions. Instead, seeking cooperation and consultation are currently one of the major activities of HCP/VE in the care for patients with SUD+ in a palliative care phase.

**Male*:*** *I don’t think it’s terrible if I don’t know something, but I find it terrible if I can’t call anybody who does know. I am desperately looking for that* […] **Male:** *I agree. If you have a patient like this, then we should have a consultation like this* [the focus-group]*.* […] *How are we going to agree on things? Who is available for what questions?* [...] *we don’t use each other’s expertise.*

- Group IV (southern part of the Netherlands)

As a result of knowledge specialization and compartmentalization, hardly any care settings are available where both high-quality palliative and high-quality addiction care meet. Consequentially, HCP/VE often seek for the most appropriate care-settings. Moreover, stigma can result in denying care for or admission of these patients. Because patients prefer freedom and independency, they themselves, too, often are reluctant. As a result, patients are frequently moved from one setting to another before final admission to a care setting. Such final places can be existing care-settings or specialized care-settings, e.g. special residencies for patients with active substance use. These latter, though, are not structurally available throughout the country.

*Hospices are critical if you present patients like this. However, before we do so, we are even more discerning, like ‘is it possible for this person to go to there? Does he suit that social environment?’*

- Male, group I (southern part of the Netherlands)

#### Content of care

HCP/VE reported that daily practice of caring for this patient group comprehends (ethical) dilemmas on substance use and abstinence. On both issues, no universal or univocal policies within and between institutions exist. As a result, pain too, allows for an ethical issue. Due to active or secret substance use and the interaction with and habituation to medicines, analgesics and sedatives often appear insufficient and knowledge inadequate. Even with high dosages, patients suffer from a lot of pain. HCP/VE working in PC settings called themselves “givers” and they, for this reason, struggle with setting boundaries.

*We are trying to provide quality of life and meet someone’s wishes as much as possible* […]. *Yeah and well, these people often only want a shot or whatever. We can’t give morphine all the time. That’s a dilemma.*

- Female, group IV (southern part of the Netherlands).

HCP/VE in daily practice furthermore, are involved with family-members. These latter often have difficult histories with patients and are in great need of emotional support. HCP/VE find it very important to care for them but do not always find the time to do so. Sometimes, volunteers fulfill this role.

**Female:** *The Salvation Army has a special association for this* […]. **Female:** *People who try to find them* [family members] *and have conversations and so on.* **Female:** *Yes. And also, they provide support afterwards, because often there is … You didn’t lose touch just like that.*

- Group II (central part of the Netherlands)

Another group of proxies that was mentioned, concerns co-patients with SUD+. Some HCP/VE proposed to increase their involvement in PC as they know a patient for a long time and know their preferences for contact, communication and care. However, most patients often have no or very small social networks and consequentially, HCP/VE sometimes carry more responsibilities, e.g. in making end-of-life decisions.

#### Delivery of care

HCP/VE considered creativity important to relate to compartmentalization of healthcare, communication with patients, the lack of structural knowledge and appropriate care-settings. To provide the most optimal care achievable, tailored solutions are sought for. Exceptions and “unconventional” care practices seem common and universal policies seem impossible. HCP/VE need to be flexible and accepting, e.g. in deviating from regular medication frameworks. Care is often delivered in a practice- instead of evidence-based fashion.

*Well, it is … Educated guesses. We don’t act just like that. It is experienced-based* […]. *With each following case, I can see we are doing better. So from that point of view, it is indeed ‘trial and error’ and learning from that.*

- Male, group I (southern part of the Netherlands)

In addition, several attitudes and values were regarded important in the communication with this patient group. First, in view of patients’ tendency of care avoidance, secrecy and their closed communication, HCP/VE need to be very active, persistent and alert in reaching and meeting patients, getting information and signaling symptoms. Proactive structural medical checks and keeping care goals low are ways to handle this. Second, HCP/VE told that having time and respect and being non-judging and non-hierarchical are important to connect and develop a trusting relationship with a person.

*… trust isn’t obvious.* […]. *So, you really have to find that connection, even when your own background is totally different.*

- Male, group II (central part of the Netherlands)

### Outcome

Patient Characteristics, the Structure and Process of PC for patients with SUD+ delineate the Outcome of care. Three outcomes are found.

First, HCP/VE stated that often the PC phase of patients with SUD+ is not recognized in a timely manner as a consequence of limited communication and a lack of cooperation and knowledge. Providing proactive care appears hard and HCP/VE stated that the range of the surprise question (“would I be surprised if this patient would die within 12 months?”) is inappropriate as for too many patients with SUD+ the answer would be “no”. Also, timely identification is complicated by the nature of and the unpredictable care-trajectories of the diseases that patients often suffer from, such as COPD. Due to late identification, disease can be far-advanced and patients could die acutely or have a short terminal care phase only. HCP/VE stated that they feel unprepared and that care-trajectories are very hasty.

*What we struggle with is the identification of that phase. Because actually, we want to anticipate on good palliative care and that appears to be very hard for this target group.*

- Female, group III (central part of the Netherlands)

On the other hand, HCP/VE are very surprised about the length of time some patients still live after identification of the need for PC and receiving this care. They stated that several factors might explain the unexpected length of time. For one, the identification of the PC phase forms a starting point of the delivery of more integrated care, resulting in a stabilization of disease. It has also been suggested that these patients are “fighters” or that abstinence has positive effects on health. HCP/VE agreed that such a prolonged PC phase provides opportunity to improve quality of life (QoL) and to organize e.g. family reunion.

The second Outcome concerns a desire for an expertise team for both consultation as well as provision of PC at the most appropriate place. This could not only increase patients QoL, but also mutual understanding between palliative and addiction care and feelings of competency. HCP/VE, however, are worried about is feasibility.

**Female:** *I am just like ‘could the consult function be free of charge?’. I could give so much advice, I could come over, I could mean so much and have a look together, but always it is ‘it is not paid for, it is not financed’. We should make it claimable.*

- Female, group VI (western part of the Netherlands)

Finally, since it can be demanding to provide PC, to communicate with this patient group and to provide care in the quite limited structure of care, HCP/VE deemed peer supervision and care-evaluations very important. Care for the caregivers, however, appears underdeveloped due to time-restraints.

### SWOT-framework

**Fig. 2:**
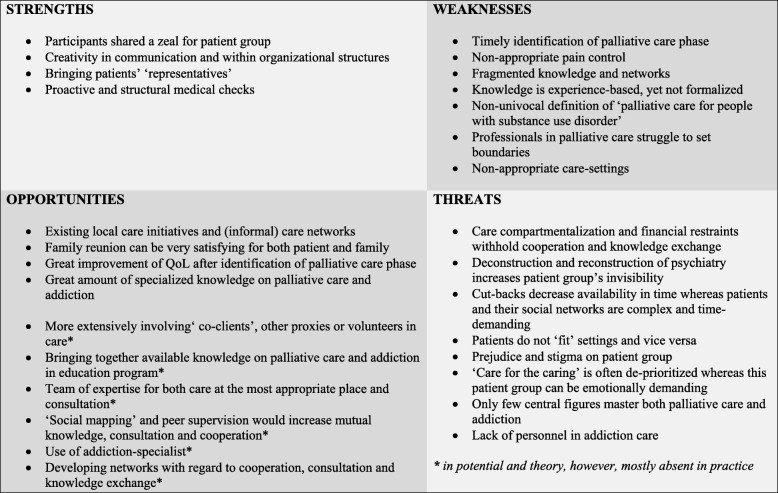
Strengths, Weaknesses, Opportunities and Threats (SWOT) of palliative care for patients with substance use disorder and multiple problems

Figure 2 serves as a summary and explication of Strengths, Weaknesses, Opportunities and Threats of PC for patients with SUD+. It clearly shows that by targeting preconditions of care provision - namely knowledge, time and cooperation - a lot can be improved in patients’ QoL and the daily course of events for HCP/VE.

## Discussion

This study aimed to explore and collect care-experiences, opinions and views of healthcare professionals, volunteers and experts-by-experience involved in the palliative care for patients with SUD and multiple problems. We found that such care is not optimally organized and conclude that there is a lot of room for improvement. Patient characteristics (e.g. limited communication) as well as structural problems (e.g. lack of formal knowledge and time) call for a creative approach of HCP/VE towards the organization of palliative care and the patient group itself. Furthermore, professionals from addiction, palliative and general care do not cooperate optimally. PC needs of these patients are identified (too) late and they may then suffer a lot of pain. HCP/VE stated that they need more specific education and a mobile team for both consultation and care provision tailored to the patient’s needs. Recent research shows similar results [49–52]. The resources to provide optimal palliative care for patients with SUD+ appear insufficient. The need for volunteers and the Salvation Army’s involvement in this care might confirm this finding. In the context of reconstructions within the Dutch healthcare system, e.g. transitions to ambulatory care, and the ageing of population [53–55], further development of PC for this patient group is imperative.

### Practice and educational implications

In general, early PC interventions have more beneficial effects, predominantly on QoL, than standard care only or late PC alone [56–59]. For this reason, it is worrisome that the PC phase for patients with SUD+ is often identified too late. This trend is recognized within the literature for the homeless people, too [29, 31, 60]. Therefore, it might be useful to develop an identification tool for this patient group or to adapt existing tools, like the PALLI for patients with intellectual disability or the Vulnerability index for homeless people [61, 62]. Alternatively, set point(s)-of-entry to end-of-life services could be identified, e.g. harm-reduction service or hospitalization for acute exacerbations [63, 64]. Furthermore, an increased attention to Advance Care Planning for this patient group might be an option [65]. Despite patient’s denial of disease and dying, it may, for example, be revealing to confront them by asking the surprise question [66] in an opposite way: “would you be surprised if *you* died in the next 12 months?”. Such a question could be the start of exploring attitudes on and acceptance of death, dying and disease for both patients, their proxies and HCP/VE.

At times, HCP/VE were surprised about the unexpected prolonged survival and stabilization of patient’s health after the need of PC had been identified. However, one may wonder if they might have misinterpreted the patient’s health state, being misled by under treatment or untreated dying. This misinterpretation could be attributed to a lack of knowledge about PC for this patient group. HCP/VE indeed desired peer supervision or formalized education to increase their knowledge, confidence and awareness for the patient-group. The literature shows that most clinical care professionals have negative attitudes in their care for drug users or find it less satisfying than caring for other patients [67, 68]. Education, contact-based training and consultation are beneficial to change stigma and attitudes towards drug and alcohol users and confidence in providing care for them [69–72]. Also, in face of PC professionals’ inclination towards giving or even pampering, they could learn from addiction workers how to cope with addiction behavior and craving. In return, addiction workers could learn from PC professionals about this field of expertise for PC deserves to be de-associated with death and to be integrated in other systems and models of care [73, 74].

### Policy and research implications

From this study it appeared that many elements of the WHO-definition of palliative care [75] seem a bit too ideal or are challenged within the actual care practice for patients with SUD+ (see Table 3). Maybe, the two extreme values of *n* = 300 (Table 2) could be explained by lack of or confusion about a univocal definition on PC for this patient group. Particularizing this definition in future research may lead to more univocal policies, guidelines, protocols and care practice. Furthermore, it would be important to reach consensus on the meaning of PC in relation to (other) goals of addiction care, e.g. abstinence, harm reduction and stabilization [49, 76–78].

**Table 3:** Challenging the WHO definition of palliative care

“Palliative care is an approach that improves the quality of life of patients and their families facing the problems associated with life-threatening illness, through the prevention and relief of suffering by means of early identification and impeccable assessment and treatment of pain and other problems, physical, psychosocial and spiritual”.	
Part of definition	Practical challenges in PC for patients with SUD+
“their families”	There is often no social network or it is very (time- and emotionally) demanding to involve them in the provision of palliative care.
“life-threatening illness”	Patients with SUD+ can suffer from life-threatening diseases, such as cancer or COPD. However, SUD itself can be a life-threatening illness, too, but is not always recognized as such, partly because addiction care is recovery-focused. Such patients suffer from far-progressed, somatic deterioration instead of specific disease(s) and therefore, might be harder to identify as being in need of PC.
“prevention and relief of suffering by means of early identification”	Patients often suffer from a lot of pain. Since patients often still have active SUD, relief is hard. Prevention (proactive care) is challenging due to, among other things, late symptom- and disease presentation.
“impeccable assessment and treatment”	As many patients with SUD+ are limited or restrained in their expressions and experiences of symptoms and disease, assessment and treatment of pain and multidimensional problems and needs are hindered. Other barriers are that the SUD is not always known or knowledge of HCP/VE about symptoms is limited.
“other problems, physical, psychosocial and spiritual”	Caring for this patient group also comes with ethical problems.

With regard to the high amount of pain in this patient group, it is necessary to study the insufficient effect of the initiated pain treatment. Finally, it would be useful to develop a questionnaire based on the qualitative findings of this study to validate the current findings and quantify priorities in clinical practice.

### Strengths and limitations

A major strength of this study is that it was initiated from practice and, thus, is highly needed and desired by HCP/VE. Furthermore, as a result of the diversity of participants, is provides legitimate insight into clinical practice, especially when also taking into account the perspectives of patients and proxies themselves which were also studied by the authors [79]. Still, it would have been a great addition if HCP/VE from e.g. outreaching teams or emergency rooms would have been included as most participants from this study came from ‘in-patient’ settings. Also, all participants were very dedicated to the patient group, which might have biased the results.

## Conclusions

Improvements in palliative care for patients with SUD+ are greatly needed and desired by healthcare professionals, volunteers and experts-by-experience. Development of an educational program, social mapping as well as research into a univocal definition of palliative care for this patient group, would stimulate integration and cooperation between the addiction, palliative and general care sectors. Furthermore, access to resources, stigma and care for the caregivers themselves are major points of attention.

## Supplementary information

**Additional file 1.** Focus group interview guide HCP/VE.

## Data Availability

The datasets used and/or analyzed during the current study are available from the corresponding author on reasonable request.

## Abbreviations

COREQ: COnsolidated criteria for REporting Qualitative research
HCP/VE: Healthcare professionals, volunteers and experts-by-experience
PC: Palliative care
QoL: Quality of life
SUD: Substance use disorder
SUD+: Substance use disorder and multiple problems
SWOT: Strengths, weaknesses, opportunities and threats
