# Supplementary material for: Palliative care for patients with substance use disorder and multiple problems: a qualitative study on experiences of healthcare professionals, volunteers and experts-by-experience
Source: BMC Palliat Care. 2020 Jan 14;19:8. doi: 10.1186/s12904-019-0502-x (PMC6961318; doi:10.1186/s12904-019-0502-x)
Supplement: Supplementary file 1 — Additional file 1. Focus group interview guide HCP/VE. [file 12904_2019_502_MOESM1_ESM.docx]

**Date:**

**Group leader(s):**

**Focus group interview guide HCP/VE**

**Palliative care for people with substance use disorder and multiple problems**

**Introduction 10 minutes**

- Introduction of group leader(s), practical issues (e.g. duration) and goals of focus group interview and the study;
- Audio recordings, anonymity and informed consent;
- Defining palliative care (PC) and substance use disorder (SUD);
- Introduction round of participants;
- Asking for remaining questions.

**Part 1. Content of care (om several domains) 15 minutes**

- Introduction of first theme: content of care;
- If useful, link this part to relevant words of brainstorm.

1. ***When you think about palliative care for people with substance use disorder and multiple problems (SUD+), what comes to mind?***

- Brainstorm on flip-over/whiteboard;
- Clarification of / checking definitions: are participants talking about similar care?

**‘Warming-up’ 10 minutes**

1. ***Can you tell us something about your experiences with regard to providing physical care for this patient group?***

- Possible examples if conversation gets stuck: substance use (e.g. tolerance, masking of symptoms, interaction with medication), medication misuse.
- Possible prompts: dealing with these experiences, patients’ physical needs, HCP/VE problems, responsibilities, good examples, needs in physical care.

1. ***Can you tell us something about your experiences with regard to providing social care for patients with SUD+ in a PC phase?***

- Possible subjects to facilitate conversation if needed: understanding of SUD, shame, stigma, social network, reunion, unfinished business, detention, debts.
- Possible prompts: dealing with these experiences, patients’ social needs, HCP/VE problems, responsibilities, good examples, needs in social care.

1. ***Can you tell us something about your experiences with regard to providing psychological care for this patient group?***

- Possible examples if conversation gets stuck: fear, regrets and guilt, loss, loneliness.
- Possible prompts: dealing with these experiences, patients’ psychological needs, HCP/VE problems, responsibilities, good examples, needs in psychological care.

1. Explanation of spiritual (and existential) dimension by concepts like meaning giving, worldview and religion, and sources of strengths.

***Can you tell us something about your experiences with regard to providing spiritual care for this patient group?***

- Possible subjects to facilitate conversation if needed: saying goodbyes, fear, loneliness, legacy. regrets and guilt, loss, legacy, loneliness.
- Possible prompts: dealing with these experiences, patients’ spiritual needs, HCP/VE problems, responsibilities, good examples, needs in spiritual care.

**Part 2. Organization of PC for people with SUD+ 15 minutes**

- Introduction of second theme: organization of care;
- If useful, link this part to relevant words of brainstorm.

1. ***Could you give an example of a situation in which PC for this patient group was well-organized?***

- Possible examples if conversation gets stuck: responsibilities, cooperation, availability of people and resources, information transfer, place of care and death, timing and marking of PC phase, (univocal) policies about PC and SUD;
- Possible prompts: how come it was well-organized, is this recognizable for other participants?;
- ***Other examples.***

1. ***Could you give an example of a situation in which the organization of PC for this patient group was non-optimal?***

- Possible prompts: learning points, improvements, HCP/VE needs; how come it was not optimally organized, is this recognizable for other participants?;
- ***Other examples.***

1. ***What is specific about the organization of PC for people with SUD+?***

**Part 3. Communication with people with SUD+ in a PC phase 15 minutes**

- Introduction of third theme: communication;
- If useful, link this part to relevant words of brainstorm.

1. ***How do you, in general, experience the communication with people with SUD+ in a PC phase?***

- Possible subjects to facilitate conversation if needed: psychological and/or personality disorder, care avoidance and compliance, trust, openness, ethical dilemmas, decisions, involvement;
- Possible prompts: stimulating and hindering factors, HCP/VE needs, does communication differ from people without SUD+ in a PC phase?

1. ***In which way is PC attuned to the patients’ needs?***

- Possible prompts: hindering and stimulating factors, good examples, ethical dilemmas, examples.

1. ***To what extent are patients involved in planning and decisions about care?***

- Possible prompts: HCP/VE needs, difficulties.

1. ***What are your experiences with regard to the social network of people with SUD+ in a PC phase?***

**Part 4. Care for proxies 10 minutes**

- Introduction of fourth theme: care for proxies and informal caregivers;
- If useful, link this part to relevant words of brainstorm.
- Possible examples if conversation gets stuck: few/no proxies, proxies with SUD.

1. ***In what way do you take care of these proxies?***

- Possible prompt: HCP/VE needs.

1. ***How is PC attuned to proxies’ needs?***

- Possible prompts: hindering factors, good examples.

1. ***To what extent are patients involved in planning and decisions about care?***

- Possible prompts: hindering factors, good examples.

1. ***What are your experiences with the care for proxies after a patients’ death?***

- Possible prompts: good and bad examples.

**Part 5. Knowledge of and competencies within PC for people with SUD+ 15 minutes**

- Introduction of fifth theme: knowledge and competencies;
- If useful, link this part to relevant words of brainstorm.

1. ***Was the subject of people with SUD part of your education and/or additional training?***

- Possible prompts: if yes: to what extent, in which way?; If no: did you miss it?

1. ***Was there any attention to palliative care in your education and/or additional training?***

- Possible prompts: idem.

1. ***Is there additional training about these subjects in your current job?***

- Possible prompt: is there documentation available?

1. Explanation of competencies: the whole of knowledges, skills and attitudes.

***What do you think of your competence of PC for this patient group in your work setting?***

- Possible prompts: areas of (lack of) competence, cause of these feelings.

1. ***What do you think, your colleagues think of their competence in caring for this patient group?***

- Possible prompt: how come?

1. ***Within your organization, is there attention for you as a HCP/VE (‘care for the caring’) with regard to PC for people with SUD+?***

- Possible prompts: HCP/VE needs, sort of attention.

**Closing part 10 minutes**

- **Giving floor to each participants’ conclusions (e.g. main needs);**
- Providing information about declaration of travel expenses;
- Thanking participants.

**Part 6. SWOT-analysis 15 minutes**

- Explanation of goal and practical issues of SWOT-analysis (Strengths, Weaknesses, Opportunities, Threats).
